# Illuminating the role of cholinergic signaling in circuits of attention and emotionally salient behaviors

**DOI:** 10.3389/fnsyn.2014.00024

**Published:** 2014-10-27

**Authors:** Antonio Luchicchi, Bernard Bloem, John Noel M. Viaña, Huibert D. Mansvelder, Lorna W. Role

**Affiliations:** ^1^Department of Integrative Neurophysiology, Center for Neurogenomics and Cognitive Research, Neuroscience Campus Amsterdam, Vrije UniversiteitAmsterdam, Netherlands; ^2^McGovern Institute for Brain Research, Massachusetts Institute of TechnologyCambridge, MA, USA; ^3^Department of Neurobiology and Behavior, Stony Brook UniversityStony Brook, NY, USA

**Keywords:** acetylcholine, optogenetics, nicotinic receptors, limbic circuitries, attention

## Abstract

Acetylcholine (ACh) signaling underlies specific aspects of cognitive functions and behaviors, including attention, learning, memory and motivation. Alterations in ACh signaling are involved in the pathophysiology of multiple neuropsychiatric disorders. In the central nervous system, ACh transmission is mainly guaranteed by dense innervation of select cortical and subcortical regions from disperse groups of cholinergic neurons within the basal forebrain (BF; e.g., diagonal band, medial septal, nucleus basalis) and the pontine-mesencephalic nuclei, respectively. Despite the fundamental role of cholinergic signaling in the CNS and the long standing knowledge of the organization of cholinergic circuitry, remarkably little is known about precisely how ACh release modulates cortical and subcortical neural activity and the behaviors these circuits subserve. Growing interest in cholinergic signaling in the CNS focuses on the mechanism(s) of action by which endogenously released ACh regulates cognitive functions, acting as a neuromodulator and/or as a direct transmitter via nicotinic and muscarinic receptors. The development of optogenetic techniques has provided a valuable toolbox with which we can address these questions, as it allows the selective manipulation of the excitability of cholinergic inputs to the diverse array of cholinergic target fields within cortical and subcortical domains. Here, we review recent papers that use the light-sensitive opsins in the cholinergic system to elucidate the role of ACh in circuits related to attention and emotionally salient behaviors. In particular, we highlight recent optogenetic studies which have tried to disentangle the precise role of ACh in the modulation of cortical-, hippocampal- and striatal-dependent functions.

## Introduction

Acetylcholine (ACh) is essential to normal CNS function, modulating cognitive, emotional and behavioral functions, including learning and memory (Kilgard and Merzenich, 1998; Hasselmo and Giocomo, 2006), reward (Leslie et al., 2013), wakefulness and attention (Klinkenberg et al., 2011; see Picciotto et al., 2012 for a recent review). Appropriate levels of ACh are required to process relevant sensory information and for encoding environmental cues that drive goal-directed behavior (Sarter et al., 2009). Disruptions of cholinergic transmission contribute to the pathophysiology of neuropsychiatric disorders, including Alzheimer’s disease, schizophrenia and drug addiction (Court et al., 2001; Dani and Harris, 2005; Martin and Freedman, 2007). To support its prominent role in the brain, the cholinergic system sends dense projections from sparse cholinergic nuclei, that include the basal forebrain (BF), laterodorsal tegmental nucleus (LDTg), peduculo-pontine tegmentum (PPTg), and medial habenula (MHb; Woolf, 1991; Mesulam, 1995; Zaborszky et al., 1999; Ren et al., 2011). In addition, there is a small population of choline acetyltransferase (ChAT) positive interneurons in areas including the striatum and neocortex (Woolf, 1991; Mesulam, 1995; von Engelhardt et al., 2007). Cholinergic projections, from the BF, LDTg and PPTg nuclei extend throughout the main telencephalic and limbic structures delivering ACh to broad terminal fields. Released ACh activates via both ionotropic nicotinic and metabotropic muscarinic ACh receptors (nAChRs, and mAChRs, respectively) that vary in terms of cellular localization (pre- and/or postsynaptic), subunit composition, signaling mechanism(s) and affinity for ACh (for recent reviews see Wess, 2003; Gotti and Clementi, 2004; Changeux, 2010; Picciotto et al., 2012).

Although our understanding of the organization of the cholinergic system and its role in modulating certain behaviors is growing, many questions remain to be answered to understand the dynamics of ACh action and its involvement in (patho) physiology. The role of ACh in specific behaviors has been addressed using lesions of cholinergic projections or pharmacological interventions with ACh receptor activation. Such approaches, though informative, are confounded by issues of bioavailability, lack of complete reversibility and the fact that such interventions act on time scales of unknown relevance for cholinergic driven changes in excitability *in vivo*. Our understanding of how cholinergic projections innervate and modulate target circuitry remains rudimentary. In fact, it is not even clear whether ACh acts as a classic synaptic neurotransmitter—on the millisecond to tens of millisecond time scale—or whether it acts as a neuromodulator (at the hundreds of milliseconds to seconds time scale) or both (see Picciotto et al., 2012; Sarter et al., 2014). The latter hypothesis is supported by several investigations that emphasize the predominant role of ACh in modifying cell excitability and activity of entire networks of neurons (Wonnacott, 1997; Kawai et al., 2007). Moreover, a relatively modest specificity of the cholinergic system exists in terms of connectivity in crucial target regions such as the cortex (see Sarter et al., 2009, for a review). On the other hand, the presence of point-to-point sites of ACh release juxtaposed to cholinergic receptors suggests that the cholinergic system may also utilize fast synaptic signaling, typical of classic neurotransmitters (Smiley et al., 1997; Turrini et al., 2001). Indeed, the complexity of results obtained to date has led to the conclusion that ACh signaling may occur over a range of different time courses due, in part, to varied release mechanisms and proximity of release and receptive sites as well as to the involvement of distinct signaling cascades downstream of both nicotinic and muscarinic AChRs (e.g see Arroyo et al., 2014; Jiang et al., 2014). A lack of high-temporal resolution and accurate detection methods for ACh release has hampered our understanding of whether endogenous cholinergic signaling is mediated by rapid, transient release (millisecond time-scale) and/or by a more diffuse transmission (from second to minute time-scale).

With the exponential rise in the number and type of optogenetic tools developed over the last decade it is now possible to selectively stimulate or inhibit specific populations of CNS cholinergic neurons and/or their axonal terminal fields through the activation of light-sensitive opsins (for reviews see: Deisseroth, 2011; Yizhar et al., 2011; Poorthuis et al., 2014). Here, we review the recent studies that have used the expression of photo-sensitive opsins in the cholinergic system to elucidate the role of endogenous ACh signaling in different brain regions related to attention and emotionally salient/ limbic behaviors.

## Basal forebrain ACh and neocortical function

The mechanisms by which ACh release in the neocortex influences cognitive functions and behaviors are still poorly understood. While early microdialysis studies in the medial prefrontal cortex (mPFC) reported a long-lasting ACh increase during attention-related performance tasks (Passetti et al., 2000), more recent works with faster, dynamic, electrochemical detection of choline, have shown that ACh can also be released briefly in concert with cue detection in a cued appetitive response task (Parikh et al., 2007; Parikh and Sarter, 2008). Thus, while the microdialysis assays are consistent with the idea that ACh release could promote a general state of cortical arousal, due to sustained levels of ACh over long time-scales, recent and more sensitive electrochemical assays highlight a faster, and more transient release of ACh. The latter observation modifies the prior view that ACh only acts through “volume transmission” (Sarter et al., 2009), and underscores the possibility of faster components of ACh action in the modulation of specific cholinergic functions. For example, the phasic release of ACh would support more rapid transitions of cortical states, consistent with cholinergic regulation of an animal’s ability to incorporate the detection of a cue into new goal-directed behaviors (Sarter et al., 2014).

### Cholinergic fast synaptic transmission in cortex

**Figure 1:**
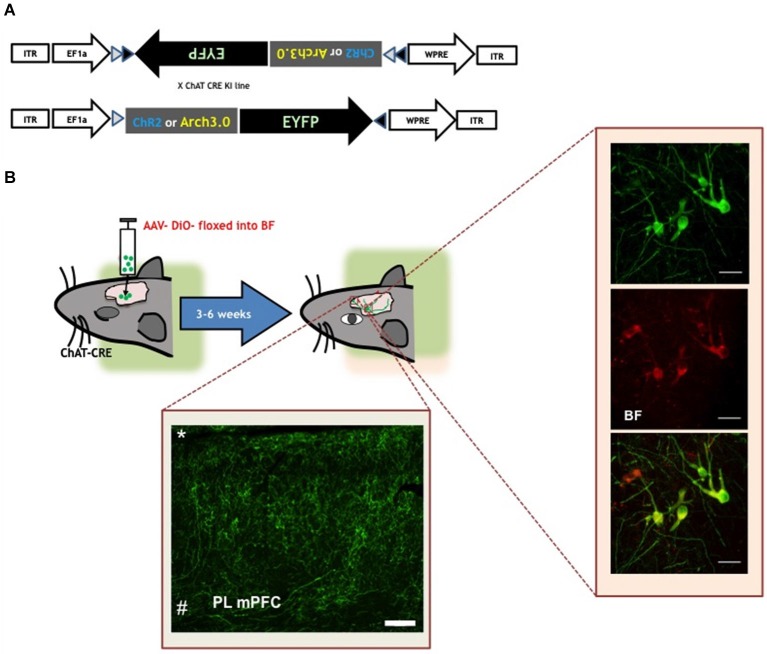
**Visualizing the cholinergic system in rodents. (A)** Viral construct used to achieve selective expression of functional opsins (ChR2 or Arch3.0) in ChAT+ cells (Witten et al., 2011). Top panel shows the construct that is sterotactically injected in the basal forebrain region of ChAT-cre mice or rats. The portion of the construct encoding the opsin and fluorophore (Enhanced yellow fluorescent protein-EYFP) is inverted and flanked by double LoxP sites (black and white triangles). After virus delivery in the brain, and in presence of cre-recombinase, the coding fragment is oriented in the right direction, allowing the expression of functional light-sensitive opsins in the ChAT+ neurons. **(B)** Schematic representation of virus delivery and opsin expression in ChAT-cre mice/rats. Rodents undergo surgery to infuse the adeno-associated virus (AAV) construct with the coding information for opsins and/or fluorophores. After 3 to 6 weeks rodents have sustained expression of the flourophore and/or excitatory (ChR2, ChIEF etc.) or inhibitory (halorhodopsin or arch 3.0) in ChAT+ cell soma and fibers. Left inset is a confocal micrograph of the basal forebrain of a ChAT-cre rat. Green cells in the top panel express EYFP as result of the AAV-floxed EYFP injection. Middle panel is a confocal micrograph of ChAT+ neurons, confirmed by the presence of anti-ChAT antibody staining. Bottom panel is a confocal micrograph indicating that the EYFP probe is expressed only in ChAT+ cells. Scale bar is 40 µm (Luchicchi and Mansvelder, unpublished observations).Bottom inset confocal micrograph of EYFP+ labeled basal forebrain terminal fields within the mPFC (*pia; # white matter). Scale bar is 200 µm (Luchicchi and Mansvelder, unpublished observations). Figure is adapted from Jiang et al. (submitted).

The application of optogenetic tools to the analysis of central cholinergic signaling using ChAT-Cre lines in either mice (Kalmbach et al., 2012; Huang and Zeng, 2013) or rats (Witten et al., 2011; see Figure 1) allows selective activation and silencing of cholinergic neurons and axonal projections, both *in vitro* and *in vivo*. Using this approach, several studies have now shown that ACh signaling occurs through direct, fast synaptic transmission—as well as over longer time scales consistent with more diffuse transmission—in the cortex (Letzkus et al., 2011; Arroyo et al., 2012, 2014; Bennett et al., 2012; Kimura et al., 2014). Activating channelrhodopsin (ChR2) in fibers from the BF elicited a barrage of inhibitory synaptic inputs to layer (L) 2/3 pyramidal cells, which depended on nAChR activation (Arroyo et al., 2012, 2014; Bennett et al., 2012; Kimura et al., 2014). Pyramidal neurons in L2/3 apparently do not express nAChRs themselves, but L2/3 interneurons do (Poorthuis et al., 2013). Activation of BF fibers produced cell type-specific responses in cortical interneurons. L1 and L2/3 LS neurons exhibited both a fast and a slow response, while L2/3 ChAT bipolar neurons exhibited only a slow response. Activation of L2/3 interneurons by ACh via both nicotinic and muscarinic receptors depressed pyramidal neuron firing thereby curtailing visual responses (Kimura et al., 2014). ACh-induced excitatory postsynaptic currents were generated by a mixed population of nAChRs (Arroyo et al., 2012). In addition to a slow dihydro-β-erythroidine (DHβE) sensitive non-α7*-mediated current, a fast component of excitatory post-synaptic potentials (EPSCs) was abolished by methyllycaconitine (MLA) in both L1 and 2/3 interneurons but not in ChAT+ cells. Comparing the reported time course of the inhibitory barrage received by L2/3 pyramidal neurons upon light-induced ACh release with the time course of the two different EPSC components, suggested that L2/3 pyramidal neuron inhibition is more likely dependent on the slow component, rather than the fast component of cholinergic activation. This was confirmed by bath application of DhβE, which prevented the inhibitory drive onto pyramidal cells. In a follow-up study, the same authors found a large trial-to trial variability of the fast component of the ACh-induced current components, indicative of direct synaptic transmission which they propose is mediated by synaptic α7*-containing (α7*) receptors. This was confirmed by lack of effect of AChE inhibitors on the amplitude or kinetics of this fast current component (Bennett et al., 2012; Arroyo et al., 2014). The slow component showed much less trial-to-trial variability and was sensitive to manipulation of AChE activity. From this, the authors conclude that the slow, non-α7* component involves diffusion of ACh over some distance, and arises from the effects of ACh on extra synaptic α4β2* nAChRs, while the faster nAChR EPSCs are mediated by direct transmission via synaptic or peri-synaptic α7* AChRs (Arroyo et al., 2012, 2014). These experiments demonstrate that in superficial layers of the somatosensory, visual and auditory cortex, L1 and L2/3 interneurons receive both direct and diffuse cholinergic inputs, that enable the cholinergic system to manipulate neocortical processing on a millisecond time scale as well as on slower time scales (Arroyo et al., 2012, 2014; Kimura et al., 2014).

On a network level, BF stimulation in anesthetized animals results in a desynchronized state of field potentials (Goard and Dan, 2009; Kalmbach et al., 2012; reviewed in Bloem et al., 2014) and neuronal firing in the BF is correlated with a reduction in low frequency, and an increase of high frequency, oscillations in the cortex (Duque et al., 2000; Manns et al., 2000). Since the frequency band activity is related to the state of arousal and the extent of cortical activation (Uhlhaas et al., 2008; Wang, 2010; Deco and Thiele, 2011; Cachope et al., 2012), this supports the idea that ACh acts as a neuromodulator involved in setting the state of arousal. Mechanistically, it was shown that ACh activated mAChRs on cortical pyramidal neurons (Gulledge et al., 2009), thereby shifting firing modes from bursting to tonic and changing low frequency high amplitude oscillatory activity to high frequency low amplitude activity on a network level (Metherate et al., 1992).

Other studies have looked at the effect of ACh on the direction of the flow of information in the cortex. Again, these studies have been performed in sensory areas because in these regions, neuronal responses could be related to sensory stimulation. In this regard it is reasonably well established that ACh is directly involved in the enhancement of feed-forward thalamic input into the sensory cortical areas (see Bloem et al., 2014, for a review). In L4 of visual cortex, ACh increases the gain and reliability of neuronal responses (Goard and Dan, 2009; Soma et al., 2012, 2013), an effect that is mediated by heteromeric nAChRs (Roberts et al., 2005; Disney et al., 2007). A similar effect of ACh is observed in the barrel cortex (Oldford and Castro-Alamancos, 2003).

In L2/3, the picture is more complex. In general, cholinergic modulation reduces firing rate in these layers by increasing GABAergic inhibition through mAChRs and nAChRs (Disney et al., 2012; Alitto and Dan, 2013; Soma et al., 2013; Kimura et al., 2014). The ACh modulation in firing rates was associated with enhancement of the reliability of encoding and modulation by stimuli presented (Goard and Dan, 2009; Soma et al., 2013).

The cortical depression associated with whisker trimming is accompanied by an increase of heteromeric nAChRs on interneurons in L2/3 and blockade of these nAChRs can prevent the cortical depression. These observations support the contention that heteromeric nAChRs in L2/3 are required for regulating the input- dependent responsiveness of the somatosensory cortex (Brown et al., 2012a).

Intra-cortical projections that connect superficial layers between different cortical columns are also inhibited by ACh through activation of mAChRs (Kimura and Baughman, 1997). Based on this finding and the reduced activity in the superficial layers, it has been suggested that ACh reduces horizontal processing through cortico-cortical interactions (Hasselmo and Giocomo, 2006). Indeed it has been observed in slices, and *in vivo* animal experiments as well as in humans, that the spatial spread of excitation in response to stimuli is reduced in the presence of elevated levels of ACh (Kimura et al., 1999; Silver et al., 2008). Such a modulation of excitation could have a sharpening effect on tuning curves of receptive fields and on discrimination of sensory stimuli (Roberts et al., 2005; Thiele et al., 2012). The combined effects of ACh—e.g., reduction of lateral interactions and increased sensitivity to thalamic inputs, would be expected to increase network sensitivity to incoming information and enhance signal to noise. A similar selective gain-control effect of ACh is observed with enhanced attention (Briggs et al., 2013) and could be one of the core mechanisms through which ACh modulates selective attention (Hasselmo and Giocomo, 2006; Deco and Thiele, 2011; Hasselmo and Sarter, 2011).

The functional impact of ACh on the deeper L5 and 6 is less well understood. It is clear that deep layer pyramidal and interneurons are modulated by both nAChRs and mAChRs (Gulledge et al., 2007; Kassam et al., 2008; Poorthuis et al., 2013). ACh is associated with both response suppression and response facilitation, although the net effect of endogenous cholinergic signaling is not clear (Soma et al., 2013). In L1, most (if not all) interneurons contain α7* and /or non-α7* nAChRs (Christophe et al., 2002; Alitto and Dan, 2013). Since these neurons inhibit both L1-3 interneurons and L2/3 pyramidal cells, the effect of cholinergic L1 activation appears to be complex with both net inhibition as well as disinhibition of pyramidal cells in deeper layers, and it is likely dependent on the source and extent of ACh release in L1 (Letzkus et al., 2011; Bennett et al., 2012; Cruikshank et al., 2012; Jiang et al., 2013; Arroyo et al., 2014).

Thalamic inputs to L5 neurons are strongly regulated by nicotinic receptor activation (Lambe et al., 2003; Couey et al., 2007; Poorthuis et al., 2013). Whether these are targeted by direct cholinergic inputs is not known. However, within the thalamic reticular nucleus, neurons receive biphasic fast cholinergic inputs mediated by non-α7* nAChRs and mAChRs (Sun et al., 2013).

### Manipulating the cortical cholinergic system during behavior

Despite new insights as to how rapidly ACh levels may rise and fall in prefrontal cortex during cue detection (Sarter et al., 2014), there is still no direct demonstration of the cellular and synaptic mechanisms by which ACh controls attentional behaviors. Hints emerge from the optogenetic data on the disinhibitory circuit mechanisms in superficial layers of sensory areas (Letzkus et al., 2011; Arroyo et al., 2014), but the architecture of the somatosensory cortex differs substantially from that of prefrontal cortical regions. Indeed, L4 is absent from rodent medial PFC (Uylings et al., 2003), and projections from the mediodorsal thalamus target all layers of mPFC, in contrast to the more discrete segregation of thalamo—cortical input seen in somatosensory areas (Douglas and Martin, 2004; Constantinople and Bruno, 2013). Few studies have appeared that manipulate the cholinergic system using optogenetics during cognitive tasks. In the primary visual cortex (V1) optogenetic stimulation of BF projections improved visual discrimination, a hallmark of visual attention, in a go-no-go task (Pinto et al., 2013). Inhibiting the BF cholinergic projections to the visual cortex with either halo-rhodopsin (NpHR) or archaerhodopsin (Arch) impaired mouse performance on the same tasks (Pinto et al., 2013; Arroyo et al., 2014 for review).

In a recent report of unpublished observations, Sarter et al. (2014) optogenetically manipulated the excitability of BF projections to the PFC in mice performing a sustained attention task (SAT). This would be the first report of optogenetic manipulation of ACh release in the PFC and modulation of attention performance. Using ChAT-Cre mice expressing ChR2 in the BF, the authors report that brief blue light stimulation during cue presentation increases detection of the cue. Optogenetic stimulation of BF fibers in the absence of a cue, which predict the presentation of reward, results in a higher number of false-positive responses in cue detection of ChR2 mice. Inhibition of ACh fibers with NpHR stimulation reduced cue detection (Sarter et al., 2014). Previous studies from the same group have identified transient release of ACh in the mPFC as a modulator of cue-directed attention. In particular, fast ACh release occurred when the cue trial was preceded by an actual or perceived non-cue trial (Howe et al., 2013). Therefore, cholinergic transients may be involved in state-shifting: i.e., in regulating the shift from generalized monitoring to one of cue-directed attention (Sarter et al., 2014). In this sense, the optogenetic increase in false-positive responses, where the animal responds incorrectly to a non-cue trial, might reveal the mechanism by which transient release of ACh in the mPFC determines the transition from cue detection to a behavioral response. Full appreciation of the data underlying these conclusions awaits publication of the primary data referred to in the Sarter review (Sarter et al., 2014).

## Optogenetic control of cholinergic projections to hippocampus and amygdala: synaptic plasticity and oscillations

Hippocampal control over specific behaviors, such as learning and memory, is potently modulated by cholinergic signaling. Antagonists to both nicotinic and muscarinic AChRs impair performance in hippocampal-dependent memory tasks in rodents (Levin et al., 2002), as well as the ability to encode spatial information (Blokland et al., 1992). The majority of cholinergic inputs to the hippocampus (up to 90%) come from the medial septum and diagonal band via the fimbria/fornix, and enter the hippocampus through the stratum oriens (SO; Frotscher and Léránth, 1985; Dutar et al., 1995). In addition, sparse cholinergic interneurons have been reported in some regions of the hippocampus, where they usually impinge on GABAergic interneurons (Griguoli and Cherubini, 2012). Both nicotinic and muscarinic AChRs are involved in regulating hippocampal network activity, such as synchronization of neuronal activity and altering of synaptic weights, thereby influencing hippocampal support of cognitive function (Yakel, 2012). Exogenous application of nicotinic agonists in hippocampal slices affects synaptic plasticity in nearly all hippocampal areas (Tu et al., 2009; Yakel, 2012), and muscarinic agonists induce fast network oscillations (Mann et al., 2005). However, it is still not completely clear how cholinergic receptors regulate rhythmic and phasic oscillations and synaptic plasticity *in vivo*, during hippocampal-dependent cognitive functions.

By stimulating septal cholinergic projecting neurons to the SO using both electrical and optogenetic methods, Gu and Yakel disentangled the temporal requirements for ACh release in the cholinergic modulation of synaptic strength of Schaffer’s collateral (SC) to CA1 synapses (Gu and Yakel, 2011). With precisely timed activation of septal cholinergic neurons in ChAT-cre mice expressing ChR2, Yakel et al. showed that when the light-evoked increase of ACh release in the SO preceded the SC stimulation by 100 ms, long-term potentiation (LTP) in the CA1 was triggered. This effect was dependent on the activation of α7* nAChRs in postsynaptic neurons. On the other hand, ChR2 activation of cholinergic terminals only 10 ms before the SC stimulation resulted in hippocampal short-term depression. In the latter case the effect was due to an α7* subunit-dependent inhibition of presynaptic glutamate release. Even more intriguing, the α7* component also altered synaptic plasticity when light pulses were delivered 10 ms *after* the SC activation. In fact, this latter protocol caused LTD in SO neurons by a mechanism which was attributed to mAChR activation, although whether the muscarinic component was pre- or postsynaptic is not clear (Gu and Yakel, 2011).

Muscarinic AChRs also modulate hippocampal activity by acting on interneurons (Bell et al., 2013). This is in line with the role of these receptors in orchestrating network oscillation within the hippocampus (Mann et al., 2005). Interestingly, interneuron network responses to light-evoked ACh release from the septum varied according to the level of cholinergic activity. In particular, low-intensity stimulation of cholinergic inputs was more likely to inhibit certain classes of interneurons via a mechanism dependent on the M4 type of mAChRs, whereas higher levels of ACh release triggered depolarization in other interneurons via broader muscarinic signaling. Cholinergic inputs from BF can also activate GABAergic interneurons through activation of α4β2* nAChRs in specific layers of the hippocampus (Bell et al., 2011).

Combining optogenetic stimulation of medial septum/diagonal band of Broca (MS/DBB) projections to the hippocampus with whole-cell patch clamp recordings and voltage sensitive dye (VSD) imaging it has been shown that inhibitory interneurons in the hippocampus receive cholinergic EPSPs in response to light stimulation of septal cholinergic fibers that are sensitive to DhβE, but not MLA (Bell et al., 2011). These light-evoked EPSPs have slow kinetics similar to the non-α7* component seen in interneurons in the somatosensory cortex (Arroyo et al., 2012). The interneurons that express α4β2* have their somata or dendrites in the SO or stratum lacunosum-moleculare (SLM) of the hippocampus. Finally, another recent optogenetic study implicates ACh release from the MS/DBB in the modulation of synaptic plasticity triggered by GABAergic interneurons of the stratum oriens lacunosum-maculare (OLM) in the SC-CA1 (Leão et al., 2012). Taken together, these data show that ACh inputs from the septum can influence hippocampal oscillations and plasticity in a highly specialized manner, resulting in a fine-tuning of hippocampal network activity in a layer specific manner and with millisecond timing. We still lack knowledge on the exact timing of activation of hippocampal cholinergic inputs during behavior. This will require both optogenetic manipulation of cholinergic projections, and concurrent visualization of activity of the BF projections in the hippocampus of awake-behaving animals.

Recently, optogenetic studies have been carried out to study the influence of other neuromodulatory systems interacting with cholinergic signaling to modulate hippocampal network activity. A set of studies conducted by Alger’s group have very elegantly demonstrated that both the endocannabinoid (eCb) and endogenous opioid systems may participate in the generation of ACh-dependent modulation of hippocampal oscillatory activity (Nagode et al., 2011, 2014). With brief stimulation of the MS/DBB fibers in the CA1, Nagode et al. (2011) reported rhythmic inhibitory post-synaptic currents (IPSCs) in pyramidal neurons, accompanied by low frequency oscillation in hippocampal slices. Interestingly, the IPSCs, which were likely evoked by interneurons impinging on the pyramidal cells, were abolished by either GABA or mAChR antagonists. Moreover, the same events were also eCb-sensitive, supporting the presence of active cannabinoid receptor (CB-R1) in the presynaptic interneuron terminal. It is widely known that CB-Rs are expressed in the hippocampus, where they drive different forms of plasticity and mediate aspects of neuroprotection (Wilson and Nicoll, 2001). Only cholecystokinin (CCK) + interneurons in hippocampus have functional CB1-Rs; CB-Rs are not present on PV+ interneurons (Katona et al., 1999). For this reason, it is likely that the cholinergic modulation of low frequency oscillations observed in this study depends solely on CCK+ cell activity. Optogenetic inhibition of either PV+ interneurons or glutamic-acid decarboxylase-2 (GAD2)+ cells in the CA1 confirmed that the PV− population of GABAergic interneurons were required for ACh induction of low frequency oscillations. Surprisingly the ability of these PV− cells to trigger low frequency rhythms in the hippocampus was blocked by a mu-opioid receptor antagonist, and subsequent induction of IPSCs in pyramidal neurons by ACh release in ChAT-Cre mice was shown to be sensitive to both CB1 and mu-receptor blockade (Nagode et al., 2014). Overall, these studies provide new insights on the possible cross-communication between the eCb and cholinergic modulatory systems in the regulation of hippocampal network activity and perhaps, in memory functions.

The effects of cholinergic input in general, and of nAChRs in particular, in the basolateral amygdala is also under study with optogenetic labeling of the neurons and projections of the nucleus basalis (Role, 2014). These studies have revealed that cholinergic signaling potently modulates the plasticity of cortical synapses on basolateral amygdale (BLA) pyramidal neurons, decreasing the threshold for induction of LTP. Excitatory effects of nucleus basalis stimulation on BLA firing is confirmed in *in vivo* recording and, most striking, the rate of extinction of responses to a cue-associated fear conditioning paradigm is slowed by brief optogenetic activation of the cholinergic terminal fields in BLA during training (Role, 2014). These findings are consistent with the idea that cholinergic signaling reinforces amygdala-based memories, perhaps rendering them less susceptible to subsequent extinction (Role, 2014).

## Modulation of striatal circuits by ACh

### Cholinergic interneurons modulate the release of multiple striatal neurotransmitters

In addition to the robust modulatory activities of cholinergic signaling in cortex and hippocampus ACh is renowned for its strong regulatory role in subcortical brain regions within the midbrain and striatum. In particular, the core of the brain reward circuitry, comprising the ventral tegmental area (VTA) and the nucleus accumbens (NAc), is strongly modulated by ACh. The main source of ACh to the VTA neurons in the midbrain arises from the brainstem structures LDTg and PPTg, which play a role in acquisition of reward, and reward-related locomotor activity (Corrigall et al., 2002; Champtiaux et al., 2006). The main source of ACh for the NAc/ventral striatum, as well as for the dorsal striatum, is the cholinergic interneurons which comprise less than 2–5% of the total striatal neuron population (Descarries et al., 1997). Notwithstanding the paucity of striatal cholinergic interneurons, ACh signaling is directly involved in the modulation of (1) striatal dopamine (DA) release (Rice and Cragg, 2004; Exley and Cragg, 2008; Wang et al., 2014); (2) local network functionality (Galarraga et al., 1999; Koós and Tepper, 2002); and (3) striatal-dependent behaviors related to reward (Joshua et al., 2008).

The release of DA in striatum is crucial for functions such as motivation, reward and locomotor activity (see Cachope and Cheer, 2014, for a recent review) and cholinergic transmission can drive striatal DA release (Exley and Cragg, 2008). A recent study showed that selective optogenetic activation of accumbal cholinergic interneurons is sufficient to trigger DA release in the same region, and that this effect is independent of the suprathreshold activation of VTA DA neurons *per se* (Cachope et al., 2012; Threlfell et al., 2012; Wang et al., 2014). As such, the activity of cholinergic interneurons might boost the release of DA to encode aspects of reward-related events. This proposal is in line with studies in which photostimulation of cholinergic interneurons drove striatal DA release via activation of presynaptic nAChRs (Threlfell et al., 2012; Wang et al., 2014). On the other hand, Cachope et al. (2012) reported that the direct effect of cholinergic interneuron activation on DA release was only partially mediated by activation of AChRs. Combining optogenetic manipulation with *in vitro* pharmacology, revealed the collaboration of both nicotinic (β2*) and muscarinic receptors, together with the activation of α-amino-3-hydroxy-5-methyl-4-isoxazolepropionic acid (AMPA)-type glutamate receptors in the enhancement of striatal DA release. Thus, a synergy exists between ACh and glutamate in modulating the activity of the striatal network. The source of glutamate may be the striatal cholinergic interneurons themselves (Gras et al., 2008). Activating ChR2 in cholinergic striatal interneurons triggers postsynaptic responses onto medium spiny neurons (MSNs), the most abundant striatal cell type. Under the stimulation conditions used by Higley et al., the direct postsynaptic responses were blocked by glutamate receptor antagonists alone and were insensitive to AChR blockade (Higley et al., 2011). This suggests that at low levels of stimulation direct control of MSN firing by “cholinergic” interneurons may also rely on fast glutamatergic transmission.

A follow-up of this study was conducted looking at the connections between ACh interneurons and other local interneurons. including the PV+ interneurons, that also contact MSNs directly (Koós and Tepper, 1999; Gittis et al., 2010). Activating dorsal striatal ACh interneurons triggers the co-release of ACh and glutamate on PV+ interneurons, activating slow non-α7* nAChR currents, and both AMPA and N-methyl-D-aspartate (NMDA) receptors (Nelson et al., 2014). ACh and glutamate co-release was dependent on the presence of the vesicular glutamate transporter VGLUT3. This transporter is also involved in enhancing the vesicular loading of ACh and is important for di-synaptic inhibition of MSNs after PV+ excitation, a common feature in striatal information processing (Gras et al., 2008).

Activation of ChR2 in cholinergic interneurons in striatum also triggered GABA-A receptor-mediated postsynaptic currents in MSNs both *in vivo* and *in vitro* (Witten et al., 2010). Optogenetic stimulation of striatal cholinergic interneurons activated di-synaptic inhibitory responses in MSNs *in vitro* (Nelson et al., 2014). This effect was still present when PV+ neurons were ablated leading the authors to suggest that the di-synaptic inhibition of MSNs might be mediated by GABA release from DA terminals, that are studded with β2* nAChRs and targeted by cholinergic interneuronal projections. Thus, striatal network activity could be orchestrated by cholinergic interneurons through simultaneous regulation of DA and GABA release in the same striatal area.

### Optogenetic studies of role of cholinergic interneurons in striatal-dependent behaviors

Optogenetic studies have helped to define the role of striatal cholinergic interneurons in multiple aspects of motor control, associative learning and reward (see Jiang et al., 2014, for review; Exley and Cragg, 2008). During reward-related events, cholinergic interneurons initially increase their firing activity, and then pause, after which they start firing in a third phase of elevated activity (e.g., Morris et al., 2004). Most likely, these phasic activity periods support DA release from VTA projections, with the nAChR mediated component being *independent* of VTA action potential firing (e.g., see Wang et al., 2014). By combining pharmacological, optogenetic and electrophysiological techniques, Straub et al. (2014) recently suggested reward coding resides in the pause in striatal ACh interneuron activity that results from the direct effect of nigrostriatal DA projections via D2 dopamine receptors on cholinergic interneurons. The authors did not identify which neurotransmitter is involved in the rebound phase. Other studies have suggested that the pause of ACh interneuron firing may be caused by a GABA component. Activating VTA GABA neurons that project to the striatum with ChR2 and recording activity on ACh interneurons resulted in a pause of ACh interneuron firing (Van Bockstaele and Pickel, 1995; Tan et al., 2012; Van Zessen et al., 2012). Interestingly this effect was only observed in striatal ACh interneurons, sparing the other cell population and it was insensitive to DA receptor blockade. Behavioral studies have confirmed that GABA mediated inhibition of cholinergic interneurons is a requisite component of stimulus-outcome association under relevant learning conditions, pinpointing the pivotal role of ACh interneurons in goal directed behaviors (Brown et al., 2012b). In addition, Witten et al. (2010) have reported that cholinergic interneuron silencing by NpHR stimulation reduced cocaine preference in behaving mice.

Taken together, these findings support the idea that cholinergic interneurons play a crucial role in the modulation of striatal activity, and striatal-dependent behavior. Recent anatomical studies have also underscored the potential importance of direct projections from the brainstem (PPTg and LDTg) to striatal cells (Dautan et al., 2014). Hence, it will be interesting to learn how cooperation between these different elements of the cholinergic system modulates striatal activity.

## Summary and conclusions

The application of optogenetic tools has accelerated the acquisition of precise information about the varied modulatory and direct synaptic signaling by ACh in an array of brain regions and behaviors. Selective expression of optogenetic probes in ChAT+ neurons allows studies of the connectivity, functionality and anatomy of cholinergic neurons and circuits throughout the rodent brain (Atasoy et al., 2008; Witten et al., 2011; for reviews see: Arroyo et al., 2014; Jiang et al., 2014; Poorthuis et al., 2014). The application of these techniques has unveiled novel contributions of previously un-identified ChAT-positive neurons to activity-dependent proliferation and neurogenesis (Paez-Gonzalez et al., 2014) as well as implicating the co-storage—and perhaps co-release—of ACh and glutamate (e.g., see Higley et al., 2011). Many important challenges and new areas of exploration are now accessible to the cholinergic enthusiast. It will be particularly important to establish the precise mechanisms by which ACh modulates attention and contributes to top down executive control of directed behaviors. With the increasing number of research groups that have adopted the optogenetic toolbox, we can expect to learn more about these exciting topics in the not-so-distant future.

## Conflict of interest statement

The authors declare that the research was conducted in the absence of any commercial or financial relationships that could be construed as a potential conflict of interest.
